# Efficacy and safety of thread embedding acupuncture combined with acupuncture for chronic low back pain: A randomized, controlled, assessor-blinded, multicenter clinical trial

**DOI:** 10.1097/MD.0000000000022526

**Published:** 2020-12-04

**Authors:** Won-Suk Sung, Yejin Hong, Sae-Rom Jeon, Jimin Yoon, Eun Kyoung Chung, Hyeong Geun Jo, Tae-Hun Kim, Seungwon Shin, Hyun-Jong Lee, Eun-Jung Kim, Byung-Kwan Seo, Jieun Choi, Dongwoo Nam

**Affiliations:** a Department of Acupuncture & Moxibustion, Dongguk University Bundang Oriental Hospital, Seongnam-si, Gyeonggi-do; b Department of Clinical Korean Medicine, Graduate School; c Department of Pharmacy, College of Pharmacy; d Clinical Trial Center, Korean Medicine Hospital, Department of Korean Medicine; e Department of Acupuncture & Moxibustion, College of Korean Medicine, Daegu Haany University, Gyeongsan-si, Gyeongsangbuk-do; f Department of Acupuncture & Moxibustion, Kyung Hee University Hospital at Gangdong; g Department of Acupuncture & Moxibustion, College of Korean Medicine, Kyung Hee University, Seoul, Republic of Korea.

**Keywords:** acupuncture, chronic low back pain, randomized controlled trial, thread embedding acupuncture

## Abstract

**Background:**

Low back pain is a very common disease. Many patients with chronic low back pain (CLBP) have been treated by complementary and alternative medicine such as acupuncture (AT) treatment. A type of AT, thread embedding acupuncture (TEA), consists of a thread that can continually stimulate at the AT points and has mechanical and chemical effects. Although TEA was widely used in clinical practice, there was little evidence of its efficacy and safety for CLBP.

**Methods:**

This clinical trial was randomized, controlled, assessor-blinded, two-armed, parallel, and conducted in multiple centers. Four Korean medical institutions recruited 38 outpatients with CLBP. The participants were randomly allocated to a treatment group (TEA combined with AT) or a control group (only AT) in a 1:1 ratio. All participants received conventional AT twice a week for 8 weeks (16 sessions) at 15 AT points (GV3 and bilateral BL23, BL24, BL25, BL26, BL40, BL60, and EX-B5) and the treatment group participants additionally received TEA once a week for 8 weeks (8 sessions) on 10 AT points in the multifidus, spinal erector, and lumbar quadrate muscles. The primary outcome measure of this study was the change of visual analog scale (VAS) from baseline (0 week) to the end of intervention (8 weeks). Secondary outcome measures included clinically relevant improvement (minimal clinically important difference) and 3% to 50% decrease on VAS, disability level (Korean version of Roland and Morris disability questionnaire), quality of life (Korean version of European quality of life 5dimension), global assessment (patient global impression of change), economic analysis, credibility test, and safety assessment.

**Results:**

The treatment group showed a significant reduction in VAS scores when compared with the control group (–33.7 ± 25.1 vs –15.6 ± 17.0, *P* = .013). As for the secondary outcome measures, the treatment group showed significant difference in 50% decrease on VAS and patient global impression of change. There was no serious adverse event associated with TEA and AT.

**Conclusion:**

This clinical trial documents the efficacy and safety of TEA combined with AT for the management of CLBP.

## 1 Introduction

Low back pain (LBP) is a very common disease and 80% to 85% individuals experience it at some point in their lives.^[1]^ According to the duration of its occurrence, LBP can be classified into; acute (<6 weeks), subacute (6–12 weeks), or chronic (>12 weeks).^[2]^ Among them, chronic low back pain (CLBP) results not only in functional disability but also socioeconomic burden^[3]^ and decreased quality of life. For patients of CLBP, continuous treatment and preventive measures are required.^[4]^

While surgical treatment of CLBP is not widely supported,^[5]^ non-steroidal anti-inflammatory drugs (NSAIDs) are suggested to be effective for short-term symptomatic relief in acute LBP patients.^[6]^ However, NSAIDs are not recommended for long-term use^[7]^ as they have been reported to have little analgesic effects with a possibility of adverse events in treating CLBP.^[8]^ These disadvantages in conventional treatment have led to many patients turning to complementary and alternative medicine (CAM).^[9,10]^

In CAM, acupuncture (AT) has been widely used as a treatment for musculoskeletal pain,^[11]^ and several clinical trials^[12]^ and meta-analyses^[13]^ have reported its therapeutic effects on CLBP. In recent studies, AT has been recognized as a dependable method for the treatment of CLBP based on high quality guidelines and also been recommended by trials comparing AT to other therapeutic interventions.^[14,15]^

Thread embedding acupuncture (TEA), involving a special type of medical thread insertion, has been widely used for the treatment of musculoskeletal pain.^[16]^ The thread used in TEA gradually softens and dissolves with time in the subcutaneous tissue or muscle.^[17]^ These processes might induce mechanical and chemical stimulation in these muscles. TEA could stimulate and exert a retention effect in the insertion points with aseptic inflammatory response, ultimately resulting in the tissue recovery promotion ultimately.^[18]^

Lack of treatment for a long duration might increase the risk of recurrence and exacerbation of CLBP. Due to the above-mentioned reasons, TEA is expected to be effective and suitable for CLBP patients who cannot receive continuous treatment. However, there have hardly been any clinical studies that confirmed the efficacy and safety of TEA for CLBP. Therefore, we conducted this clinical trial to investigate the effect of TEA combined with AT for CLBP.

## 2 Methods

### 2.1 Study design

This study was a randomized, assessor-blind, multicenter clinical trial with 2 parallel groups, and was conducted at 4 Korean medical institutions (Kyung Hee University Korean Medicine Hospital, Kyung Hee University Korean Medicine Hospital at Gangdong, Dongguk University Bundang Oriental Hospital, and Oriental Medicine Hospital of Daegu Haany University).

### 2.2 Ethics approval

This study was conducted in accordance with the Helsinki Declaration and was approved by Institutional Review Boards (IRB) of the 4 hospitals (IRB No.: KOMCIRB-160919-HR-050–13, KHNMC OH 2016-10-012, DUBOHIRB-2016-0013, DHUMC-D-16013-ANS-02). This study was also registered at the Clinical Research Information Service (CRIS, KCT0002666).

### 2.3 Sample size calculation

There were no previous studies that investigated the efficacy of AT combined with TEA. Under the assumption that the synergic effect of the combination treatment (TEA + AT) would be insignificant, we studied the results of randomized control trial that applied 4 week-treatment of TEA or sham TEA to CLBP patients.^[19]^ In this study, the changes of visual analog scale (VAS) between the baseline and intervention completion were –1.85 ± 0.86 in TEA group and –0.5 ± 0.80 in sham TEA group. Using these data, we calculated that we would require 11 participants per group with 95% power (1–*β*) at the 0.05 *α* level of significance. Considering a 40% dropout rate, we calculated the final number of each group as 19, and a total of 38 patients were enrolled.

### 2.4 Study participants

The 38 patients with CLBP were recruited for the study by predefined inclusion and exclusion criteria. All patients were 19 to 65 years old, had LBP as a chief complain for >3 months, and had over 40 mm/100 mm VAS pain and bothersomeness. Patients were excluded if they had hypersensitive reactions to AT or TEA treatment. Individuals were excluded if they: had a history, of abnormalities in the lower extremity neurological examination, needed spinal surgical treatment by severe neurological deficits, had specific disease diagnosis including; vertebral fracture, inflammatory spondylitis, spinal infection, malignant tumor neuromuscular scoliosis, or neurodegenerative diseases. All patients were informed about the study design, expected benefits, risks, and that they were able to withdraw the study at any visit. They also voluntarily consented to the study, after which, the investigator performed examinations and assessed their eligibility.

### 2.5 Randomization

A random sequence was generated with R 3.4 blockrand package (R Core Team, 2017) by an independent clinical researcher who was not involved in this trial. After the eligibility assessment, participants were randomly allocated to the treatment or control group (1:1 ratio). A random code was delivered in the sealed envelopes and kept in a double locked cabinet. A clinical research coordinator (CRC) at each site opened the envelope in sequence and allocated a participant to each group, after all the screening criteria were satisfied. The randomization code was kept by an independent statistician during the trial period and concealed until the occurrence of an event predefined as a reason for cancellation.

### 2.6 Blinding

The additional TEA meant that the participants and practitioners could not be blinded. Thus, the practitioners who performed TEA did not assess the outcome. The assessor was instructed to ask simple questions and write in detail to record the case report form, preventing the assessor and participants from talking about the treatment. The data collector and statistical manager were also blinded for this study.

### 2.7 Interventions

#### 2.7.1 Study schedule

**Figure 1:**
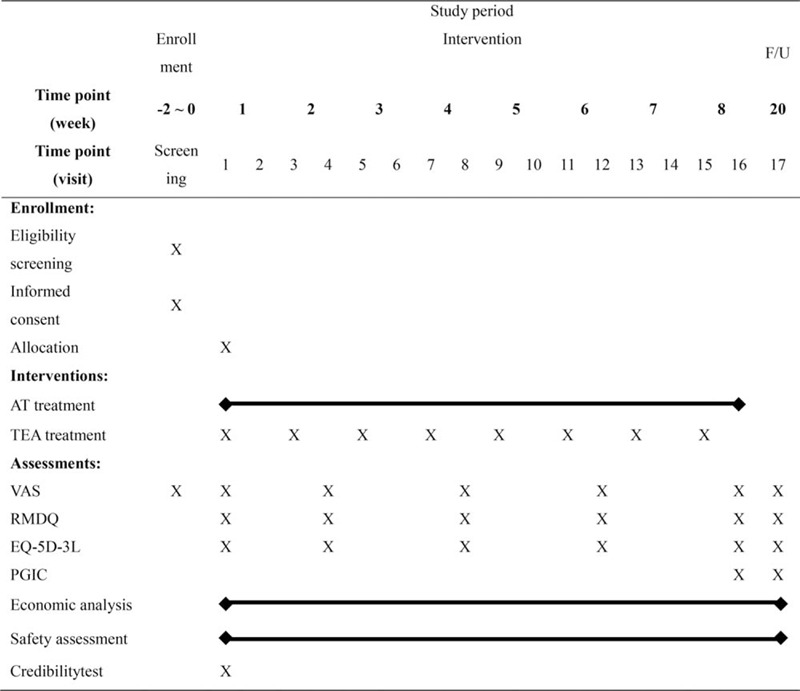
Study schedule. AT = acupuncture, EQ-5D-3L = European quality of life 5dimension, F/U = follow up, PGIC = patient global impression of change, RMDQ = Roland and Morris disability questionnaire, TEA = thread embedding acupuncture, VAS = visual analog scale.

At the screening visit, patients who were interested in participation visited one of the four Korean medical institutions and signed the informed consent form. After this, the investigator performed several examinations including physical examinations, checking vital signs, lumbar spine x-ray, blood chemistry test, demographic data, CLBP and other disease history, concomitant treatment data, and VAS to determine the patient's eligibility. If participants were eligible, the investigator determined the initiation of treatment according to the medication history. If the participant had taken anti-inflammatory or analgesics at the time of the visit, the investigator gave them a 2-week washout period. During the 8-week treatment, participants in both the groups visited the hospital twice a week (total 16 visits) and received AT at every visit. Additionally, participants in the treatment group received TEA once a week. After visit 1, participants in both groups were asked to answer the survey every 2 weeks (at visit 4, 8, 12, and 16). A final assessment was conducted on all participants at visit 17, 3 months after the last treatment (Fig. 1).

#### 2.7.2 AT treatment

The AT treatment was conducted identically in both groups. Participants lied in the prone position and received AT by sterilized stainless steel needles of 0.25 mm width and 40 mm length (DB108C; Dongbang Medical Co., Boryung-si, South Korea) at 11 lumbar-local and 4 distal AT points for 20 minutes. Lumbar-local AT points were GV3 and bilateral BL23, BL24, BL25, BL26, and EX-B5 while distal AT points were bilateral BL40 and BL60. Korean medical doctors, who were specialists in AT and moxibustion, conducted the AT treatment.

#### 2.7.3 TEA treatment

**Figure 2:**
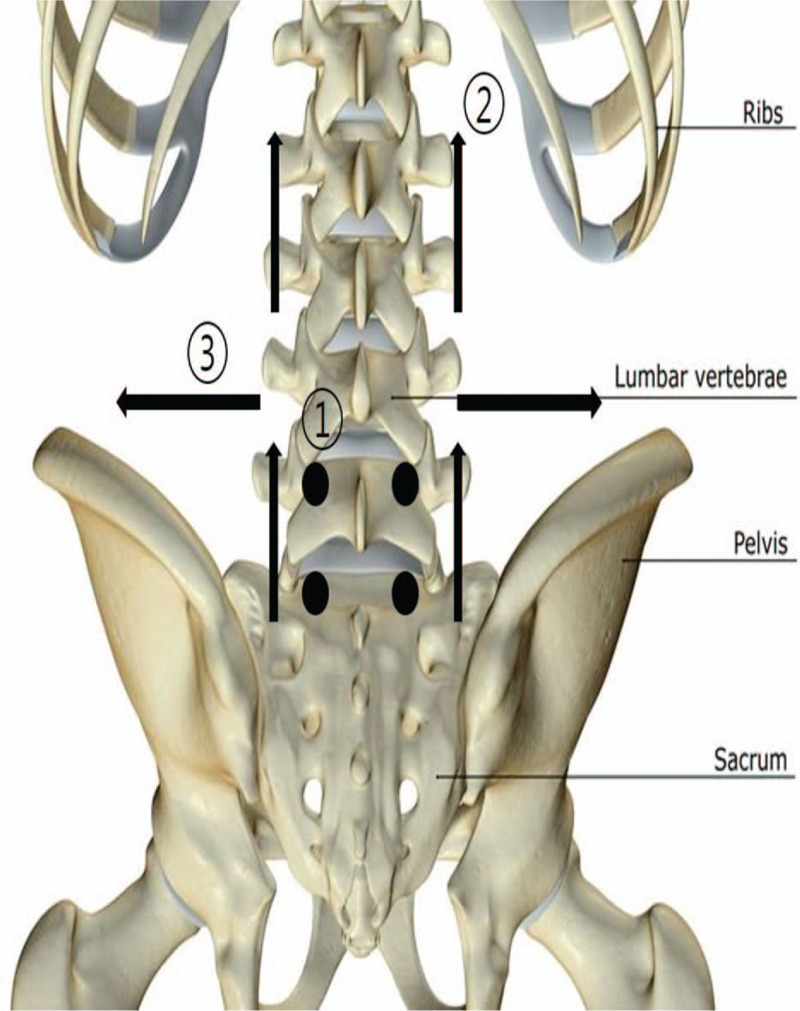
Point location and direction of TEA. TEA = thread embedding acupuncture.

Participants in the treatment group lied in the prone position and received TEA treatment by polydioxanone sutures of 29 gauge and 40 mm length (Hyundae Meditech Co., Weonju, South Korea) on the muscles of the lumbar region. TEA was performed with, a 4 cm perpendicular insertion at the bilateral EX-B2 of the L4–5 and L5–S1 for multifidus muscle stimulation, a 4 cm transverse insertion at the bilateral 3 to 4 cm of L3 and S1 spinous processes toward L1 for spinal erector muscle stimulation, and a 4 cm transverse insertion at the bilateral 3 to 4 cm of L4 transverse process toward iliac crest for lumbar quadrate muscle stimulation (Fig. 2). Ten threads were located in the shallow muscle layers. Participants received the TEA by Korean medical doctors who were specialists in AT and moxibustion.

#### 2.7.4 Concomitant treatment

Participants were allowed to continue to consume concomitant medications taken over 4 weeks before trial participation or those that were considered to have no effect on the results of this study. This was assessed by investigators. Some medications including muscle relaxants, NSAIDs (including those used for topical application or patches), antidepressant agents, and anticonvulsants or Korean medicine treatments (AT, herbal medicine, and cupping), physical therapy, injections, and surgery were not permitted during the treatment period.

### 2.8 Outcome measurement

#### 2.8.1 Primary outcome

The change of visual analog scale (VAS)^[20]^ between baseline (visit 1) and intervention completion (visit 16) was the primary outcome measure. Indicating 0 mm as the absence of pain and 100 mm as the worst pain imaginable, the participants were asked to indicate the degree of pain and bothersomeness by LBP.

#### 2.8.2 Secondary outcomes

Along with the change of pain (VAS), clinically relevant improvement was assessed using the minimal clinical important difference (MCID). Based on previous studies, MCID in CLBP is defined as a 20 mm decrease on VAS.^[21]^ The proportion of participants who showed >20 mm decrease on VAS, and >30% or 50% decrease on VAS between baseline and visit 4, 8, 12, 16, and 17 was recorded.

The Roland and Morris Disability Questionnaire (RMDQ) was used for disability level assessment.^[22]^ Zero indicated no disability and 24 indicated maximum disability, the change of the RMDQ between baseline and visit 4, 8, 12, 16, and 17 was compared.

Quality of life was assessed by the 3-level version of European quality of life 5dimension (EQ-5D-3L).^[23]^ Each dimension, mobility, self-care, activities of daily life, pain, and anxiety/depression, was evaluated on a 1 to 3 scale. In addition to EQ-VAS that assessed the current health state, the change of the EQ-5D between baseline and visit 4, 8, 12, 16, and 17 was compared.

For global assessment, patient global impression of change (PGIC)^[24]^ was used at visit 16 and 17. With a 7-point scale indicating 1 as completely recovered and 7 as vastly worsened, participants’ impression of the change was assessed and we considered 1 to 2 as improved, 3 to 5 as unchanged, and 6 to 7 as deteriorated.

For economic analysis, cost data was collected by medical cost (including official costs such as payments for treatment and non-official costs including dietary supplements, medical devices, and orthotic device) and non-medical cost (including the costs of transportation, case, and time). In addition, a credibility test about expectancy of treatment was conducted by administering a questionnaire^[25]^ and safety assessment about adverse events occurrence ratio was calculated and compared each group.

### 2.9 Statistical analyses

The statistical analyses were performed by the principle of intent-to-treat analysis, and missing values were replaced by the last observation to carry forward the analysis. Baseline characteristics included demographic and clinical data. Continuous variables were expressed as means ± standard deviation (SD) and categorical variables were expressed as frequency and percentage.

For continuous variables of primary and secondary outcome measures, a paired *t* test or the Wilcoxon signed-rank test was used for comparisons within each group, and independent 2-sample *t* test or the Wilcoxon rank-sum test was used to compare the groups. For the categorical variables of secondary outcome measures, Chi-square test or Fisher exact test was used to compare the groups. In the safety analysis, the frequency and ratio of adverse events was applied and compared between the 2 groups using Chi-squared test or Fisher exact test. Statistical tests were performed with a significance level of .05.

A cost-effectiveness analysis (CEA) was performed to compare the cost-effectiveness of the AT with or without TEA. Due to the short cycle (20 weeks) of the intervention and outcome measure, we utilized a decision tree model^[26]^ using TreeAge Pro software (TreeAge Software, Inc., MA). For constructing a decision tree model, we needed to set treatment success and quality-adjusted life years (QALY).^[27]^ Treatment success was defined as 20 mm decrease on VAS at visit 16, based on the MCID. QALY was calculated using EQ-5D-3L score at each of the 6 visits, (visit 1, 4, 7, 12, 16, and 17) based on the previous studies.^[28]^

## 3 Results

**Figure 3:**
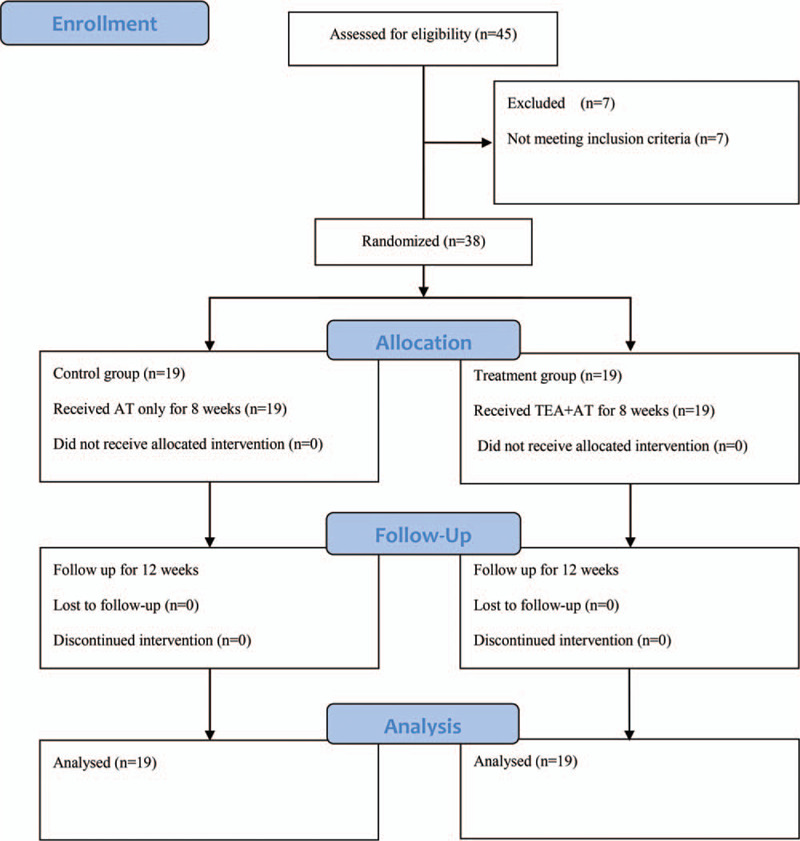
CONSORT flow diagram. AT = acupuncture, TEA = thread embedding acupuncture.

We recruited 45 patients with CLBP from August 16, 2017 to May 22, 2019 and enrolled 38 patients who met the eligibility criteria. These 38 patients were randomly allocated to the treatment or control group in a 1:1 ratio. The study had no dropouts and all of 38 patients completed the study (Fig. 3). There were no significant differences in demographic data including age, sex ratio, height, and body weight, and CLBP-related data including VAS, RMDQ, and EQ-5D (Table 1).

**Table 1:** Baseline characteristics and outcome measures of participants.

	Group		
Variables	TEA + AT (n = 19)	AT (n = 19)	*P*-value
Age, y	45.6 ± 14.6	44.1 ± 14.1	.683^a^
Gender (n, %)			
Male	5 (26.3%)	8 (42.1%)	.494^b^
Female	14 (73.7%)	11 (57.9%)	
Height, cm	163.6 ± 6.2	165.1 ± 8.1	.542^a^
Weight, kg	65.8 ± 10.8	64.7 ± 9.3	.733^a^
VAS	63.4 ± 11.9	59.5 ± 11.3	.309^a^
RMDQ	8.4 ± 3.3	8.1 ± 4.3	.528^c^
EQ-5D	0.61 ± 0.16	0.60 ± 0.17	.309^a^
EQ-VAS	60.2 ± 16.5	55.6 ± 14.9	.370^a^

AT = acupuncture, EQ-5D = European quality of life 5dimension, RMDQ = Roland and Morris disability questionnaire, TEA = thread embedding acupuncture, VAS = visual analog scale.

a Independent 2 sample *t* test.

b Chi-squared test.

c Wilcoxon rank sum test.

### 3.1 Primary outcome measure

Each group had statistically significant reductions in the change of VAS from baseline (0 week) to visit 16 (8 weeks, *P* < .001). The decrease in VAS in the treatment group was significantly greater than that in the control group (–33.7 ± 25.1 vs –15.6 ± 17.0, *P* = .013) (Table 2).

**Table 2:** Primary and secondary outcome measures at visit 1, 4, 8, 12, 16, and 17.

Variables	Group	Visit 1	Visit 4	Visit 8	Visit 12	Visit 16	Visit 17
VAS	TEA + AT (*Δ*) *P*-value^a^	63.4 ± 11.9	61.1 ± 11.6 (–2.3 ± 12.2) .428	45.5 ± 14.0 (–17.8 ± 18.9) <.001^∗∗∗^	39.9 ± 17.4 (–23.5 ± 25.6) <.001^∗∗∗^	29.6 ± 21.1 (–33.7 ± 25.1) <.001^∗∗∗^	28.4 ± 21.2 (–35.0 ± 26.6) <.001^∗∗∗^
	AT (*Δ*) *P*-value^a^	59.5 ± 11.3	55.5 ± 11.9 (–4.0 ± 12.5) .179	55.0 ± 13.3 (–4.5 ± 8.8) .039^∗^	46.5 ± 15.3 (–12.9 ± 14.4) <.001^∗∗∗^	43.9 ± 17.3 (–15.6 ± 17.0) <.001^∗∗∗^	36.2 ± 22.3 (–23.3 ± 21.2) <.001^∗∗∗^
	*P*-value^b^	.309	.666	.010^∗^	.13	.013^∗^	.141
MCID	TEA + AT		2/17	10/9	11/8	13/6	14/5
	AT		2/17	0/19	7/12	7/12	12/7
	*P*-value^b^		1.000	.001^∗∗∗^	.33	.104	.727
More than 30% decrease on VAS	TEA + AT		1/18	9/19	11/8	14/5	14/5
	AT		2/17	1/18	8/11	9/10	15/4
	*P*-value^b^		1.000	.01^∗∗^	.516	.184	1.000
More than 50% decrease on VAS	TEA + AT		0/19	6/13	9/10	13/6	13/6
	AT		0/19	0/19	2/17	4/15	7/12
	*P*-value^b^		NA	.026^∗^	.032^∗^	.009^∗∗^	.104
RMDQ	TEA + AT (*Δ*) *P*-value^a^	8.4 ± 3.3	7.1 ± 3.7 (–1.4 ± 2.5) .027^∗^	6.1 ± 3.6 (–2.3 ± 2.6) <.001^∗∗∗^	4.8 ± 3.8 (–3.6 ± 3.8) <.001^∗∗∗^	4.7 ± 4.0 (–3.7 ± 2.9) <.001^∗∗∗^	4.0 ± 4.1 (–4.4 ± 3.2) <.001^∗∗∗^
	AT (*Δ*) *P*-value^a^	8.1 ± 4.3	7.2 ± 3.4 (–0.9 ± 2.3) .105	6.6 ± 4.3 (–1.5 ± 2.5) .018^∗^	5.7 ± 3.1 (–2.4 ± 4.0) .019^∗^	5.2 ± 3.3 (–2.8 ± 4.2) .009^∗∗^	4.6 ± 3.7 (–3.4 ± 4.2) .002^∗∗^
	*P*-value^b^	.528	.544	.316	.347	.45	.414
EQ-5D	TEA + AT (*Δ*) *P*-value^a^	0.611 ± 0.163	0.650 ± 0.111 (0.040 ± 0.127) .221	0.671 ± 0.101 (0.060 ± 0.136) .055^∗^	0.735 ± 0.128 (0.124 ± 0.215) .005^∗∗^	0.736 ± 0.167 (0.126 ± 0.221) .023^∗^	0.758 ± 0.186 (0.147 ± 0.244) .011^∗^
	AT (*Δ*) *P*-value^a^	0.603 ± 0.172	0.594 ± 0.187 (–0.009 ± 0.115) .731	0.640 ± 0.172 (0.037 ± 0.110) .169	0.677 ± 0.119 (0.074 ± 0.171) .071	0.699 ± 0.136 (0.095 ± 0.148) .007^∗∗^	0.734 ± 0.168 (0.131 ± 0.159) .002^∗∗^
	*P*-value^b^	.309	.220	.410	.350	.566	.930
EQ-VAS	TEA + AT (*Δ*) *P*-value^a^	60.2 ± 16.5	60.9 ± 16.2 (0.7 ± 13.5) .815	69.3 ± 10.1 (9.1 ± 15.4) .020^∗^	68.5 ± 15.7 (8.3 ± 19.0) .075	77.9 ± 8.7 (17.7 ± 18.8) <.001^∗∗∗^	71.4 ± 19.7 (11.2 ± 24.3) .061
	AT (*Δ*) *P*-value^a^	55.6 ± 14.9	58.8 ± 12.1 (3.3 ± 11.0) .234	61.4 ± 16.2 (5.8 ± 11.0) .032^∗^	63.7 ± 15.8 (8.1 ± 13.0) .014^∗^	65.6 ± 16.5 (10.0 ± 15.1) .007^∗∗^	63.3 ± 18.0 (7.7 ± 16.2) .052^∗∗^
	*P*-value^b^	.370	.531	.465	.976	.173	.613

AT = acupuncture, EQ-5D = European quality of life 5dimension, MCID = minimal clinical important difference, RMDQ = Roland and Morris disability questionnaire, TEA = thread embedding acupuncture, VAS = visual analog scale.

a Wilcoxon signed rank test.

b Wilcoxon rank sum test.

### 3.2 Secondary outcome measure

As shown in Table 2, both the treatment and control groups indicated that pain reduction increased with time, that is; as the visits and follow ups progressed. The treatment group showed more pain improvement than the control group overall, but there was no significant difference in the individual visits, except for visit 8 and 16 (visit 8: –17.8 ± 18.9 vs –4.5 ± 8.8, *P* = .010; visit 16: –33.7 ± 25.1 vs –15.6 ± 17.0, *P* = .013).

For clinically relevant improvement, the treatment group showed superior improvement than control group overall. At visit 8, above half of participants in the treatment group showed MCID, >30%, and 50% decrease on VAS and there was significant difference between groups (*P* < .05). From visit 12 onwards, participants in the control group showed increasing improvement of VAS (MCID, >30%) and there was no significant difference between the 2 groups. However, we found a significant difference in the proportion of participants with >50% VAS decrease between the 2 groups from visit 8 to 16 (*P* < .05).

For the disability level, each group had constantly and statistically significant RMDQ reductions from baseline to visit 17. The decrease of the RMDQ in the treatment group was greater than that in the control group overall (*P* < .001 vs *P* < .05), but there was no significant difference between groups.

**Figure 4:**
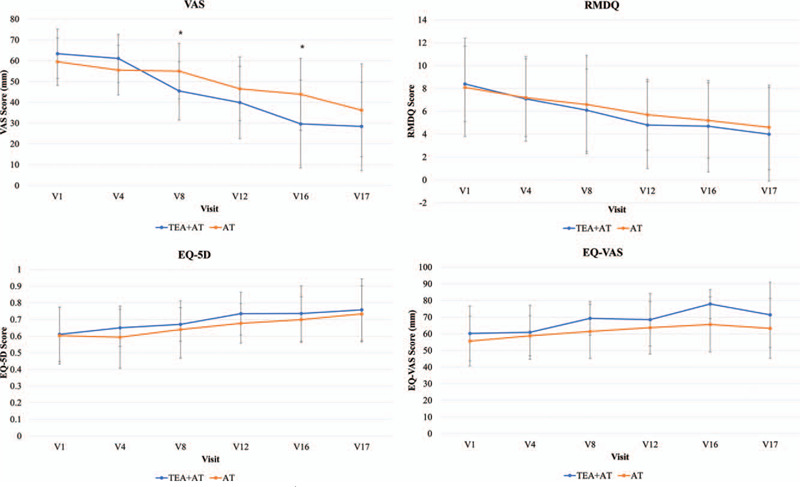
Changes in VAS, RMDQ, EQ-5D, and EQ-VAS. ^∗^Wilcoxon rank sum test. AT = acupuncture, EQ-5D = European quality of life 5dimension, RMDQ = Roland and Morris disability questionnaire, TEA = thread embedding acupuncture, VAS = visual analog scale.

For the quality of life assessment, each group had statistically significant EQ-5D improvements from baseline to visit 17. The improvement was greater in the treatment group overall and there was a worse change in the control group at visit 4, but there was no significant difference between groups. Each group also had statistically significant improvements of EQ-VAS from baseline to visit 16 (*P* < .001 vs *P* < .05), but there was no significant difference between groups (Fig. 4).

**Figure 5:**
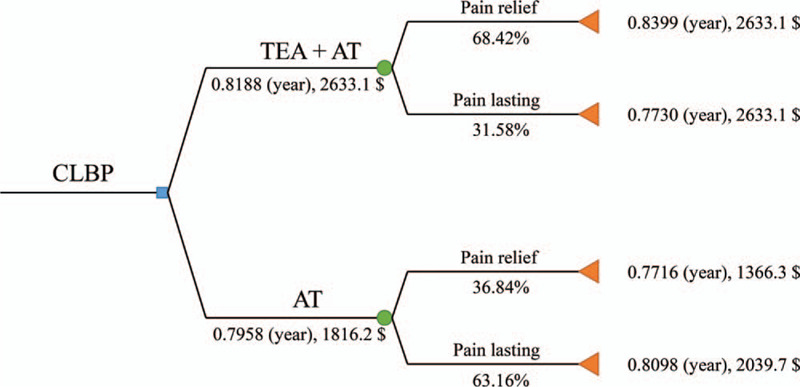
Decision Tree for economic analysis in both groups. Decision tree model showing quality-adjusted life years (QALY, year) and yearly medical costs ($) in each group. AT = acupuncture, CLBP = chronic low back pain, TEA = thread embedding acupuncture.

Regarding other secondary outcome measures, the treatment group showed significantly greater improvement of PGIC than the control group at visit 16 (*P* = .047) and 17 (*P* = .026) (Table 3). For the economic analysis, medical costs in the treatment group were higher than in the control group (Table 4). On adding the non-medical costs, total costs in the treatment group were also higher than in the control group (420.4 ± 33.2 $ vs 281.9 ± 288.9 $, *P* < .001). With total medical cost data, we utilized decision tree models and the treatment group showed higher QALY (0.8188 year) and yearly medical cost (2633.1 $) than the control group (0.7958 year, 1816.2 $) (Fig. 5). The level of credibility/expectancy was similar (7.8 ± 0.8 vs 7.2 ± 1.2) for both the groups (Table 5). For safety assessments, there were 4 adverse events in the treatment group. One case each of cystitis, allergic rhinitis, BUN increase, and type II diabetes occurred, but we determined that these events had no correlation with TEA. There were 4 adverse events in the control group. There were 3 cases of cough and 1 case of ALT increase occurred, but we assessed that these effects had no correlation with this study.

**Table 3:** PGIC at visit 16 and 17.

		Group		
Week	Scales of PGIC	TEA + AT (n = 19, %)	AT (n = 19, %)	*P*-value
Visit 16	1: Very much improved	5 (26.3%)	0 (0.00%)	.047^∗^
	2: Much improved	9 (47.4%)	11 (57.9%)	
	3: Minimally improved	4 (21.1%)	6 (31.6%)	
	4: No change	0 (0.00%)	2 (10.5%)	
	5: Minimally worse	1 (0.05%)	0 (0.00%)	
	6: Much worse	0 (0.00%)	0 (0.00%)	
	7: Very much worse	0 (0.00%)	0 (0.00%)	
Visit 17	1: Very much improved	5 (26.3%)	0 (0.00%)	.026^∗^
	2: Much improved	8 (42.1%)	6 (31.6%)	
	3: Minimally improved	4 (21.1%)	10 (52.6%)	
	4: No change	1 (0.05%)	3 (15.8%)	
	5: Minimally worse	1 (0.05%)	0 (0.00%)	
	6: Much worse	0 (0.00%)	0 (0.00%)	
	7: Very much worse	0 (0.00%)	0 (0.00%)	

AT = acupuncture, PGIC = patient global impression of change, TEA = thread embedding acupuncture.

∗ Fisher exact test.

**Table 4:** Economic analysis in control and treatment groups.

	Group		
Variables	TEA + AT (n = 19)	AT (n = 19)	*P*-value
Medical cost ($)			
Official	405.1	207.8	<.001^∗^
Non-official	0	429.3 ± 724.4	>.05
Total	405.1 ± 0	275.6 ± 290.1	<.001^∗^
Non-medical cost ($)	15.3 ± 33.2	6.3 ± 12.5	>.05
Total cost ($)	420.4 ± 33.2	281.9 ± 288.9	<.001^∗^

AT = acupuncture, TEA = thread embedding acupuncture.

**Table 5:** Credibility test and safety assessment in control and treatment groups.

		Group		
Variables	Sub variables	TEA + AT (n = 19)	AT (n = 19)	*P*-value^a^
Credibility test	Mean ± SD	7.8 ± 0.8	7.2 ± 1.2	.065
	Median	8.0	7.0	
	Min, Max	7.0, 9.0	5.0, 9.0	
Safety assessment	Adverse events occurrence (n)	4	4	1

AT = acupuncture, TEA = thread embedding acupuncture.

a Fisher exact test.

## 4 Discussion

TEA or thread embedding acupuncture is a new AT method that consists of 2 components, a guide needle and medical threads. Attached to a guide needle, the medical thread is inserted into the skin on AT or tender points. After insertion, the medical thread is known to be absorbed within 3 months^[29]^ and proceed until 180 to 210 days.^[17]^ During the absorption, TEA would produce a strong and long-term stimulation and its therapeutic effect would exert in the body. There have been several studies that reported the effects of TEA in frozen shoulder^[29]^ and osteoarthritis.^[30,31]^

In this study, we inserted TEA into the multifidus, the spinal erector, and the lumbar quadrate muscle. These muscles play a key role in the lumbar spine, and a recalled core muscles.^[32]^ In accordance with the studies that focused on the stabilization^[33]^ and strengthening exercises of core muscles^[34]^ for LBP treatment, TEA was expected to strengthen the specific muscles, resulting in pain reduction. Based on this hypothesis, we conducted our study and evaluated various outcome measures.

The primary outcome measure was VAS. This study showed better improvement of VAS between baseline and 8 weeks in the treatment group than the control group. There was an article that reported better analgesic effects of TEA than AT in the lumbar intervertebral disc herniation.^[35]^ With these results, TEA might be considered to have faster analgesic effects than traditional AT, and could be used as an attractive method in rapid pain reduction. However, this study did not show significant difference of VAS between baseline and 20 weeks between the 2 groups. More improvement in the control group during the follow up period could be a reason for a lack of statistical significance. However, absolute numbers in the treatment group did show an improvement when compared with the control group, therefore iterating that TEA + AT had better effect than AT alone.

For secondary outcome measures, the treatment group showed more improvement than the control group, but the difference was not significant. It might be considered that TEA did not show statistically superior effect while AT showed a certain effect. From a different perspective, it could be interpreted that this study identified not only approved effects of AT but also certain additional effect of TEA in diverse aspects.

Both, effect of the TEA + AT and the AT treatment alone were also found in MCID. In the treatment group, the number of participants who showed >20 mm, 30%, and 50% improvement on VAS was similar (13–14 participants). These results might mean that the proportion of early symptomatic improvement in the treatment group was high. Most participants in this group might show >50% improvement by rapid therapeutic effect of TEA and this status maintained until the 20th week follow up. In the control group, half the participants showed >30% improvement post-treatment but its proportion increased to the similar level as the treatment group at follow up, probably by persistent effect of AT.

While considering other outcome measures, the treatment group showed significant improvement in PGIC. Based on the report that PGIC focused on the patient's physical activities and mood than pain improvements,^[36]^ we might consider that the improvement of MCID and PGIC indicates both, an objective and subjective improvement of TEA. These study results also showed continuous therapeutic effects in both the groups after the follow up period. These results were similar to the results of the study by Lee et al. study.^[37]^ It might be considered that CLBP patients were satisfied with sufficient AT treatment (twice a week for 8 weeks) resulting in persistent improvement. Comparing the 20-week of this trial period to the 10-week of the previous study by Lee et al,^[37]^ we could confirm longer therapeutic effects of the treatment.

The results of the economic analysis showed that, the efficacy of the treatment group was superior to the control group, but there was no significant difference in the comparison of cost utility using QALY. In addition, the treatment group might show economic insolubility by appearing to require more yearly medical costs. Considering the method of calculating CEA, we presumed that the treatment group could not show a certain difference of EQ-5D used to calculate QALY that played a role in CEA. It would be difficult to estimate the economic evaluation with EQ-5D alone, as it would be necessary to analyze various factors to make multifaceted judgments reflecting patient preferences.

With various results from the primary and secondary outcome measures, we could found an additional beneficial effect of TEA despite a deficit of statistical significance. This study had meaningful results but there were also some limitations. First, we just evaluated the additional effect of TEA combined with AT, but not the effect of TEA alone. It might be inappropriate to suggest that TEA alone reduced the pain and improved the functional disorder. Therefore, further studies would be needed in this regard. Second, the number of each group was 19, which was close to the study by Lee et al (n = 20). A higher sample size might have increased statistical significance. More participants and a better designed trial could be needed. However, the results in this study provided a certain clinical evidence about the efficacy and safety of TEA and would be helpful for patients, physicians, and researchers in the treatment of CLBP.

## Author contributions

**Conceptualization:** Byung-Kwan Seo.

**Data curation:** Yejin Hong, Sae-Rom Jeon.

**Formal analysis:** Jimin Yoon, Eun Kyoung Chung, Hyeong Geun Jo, Tae-Hun Kim, Seungwon Shin.

**Funding acquisition:** Dongwoo Nam.

**Investigation:** Yejin Hong, Sae-Rom Jeon, Jieun Choi.

**Methodology:** Tae-Hun Kim, Hyun-Jong Lee, Byung-Kwan Seo.

**Project administration:** Hyun-Jong Lee, Eun-Jung Kim, Byung-Kwan Seo, Dongwoo Nam.

**Resources:** Seungwon Shin.

**Software:** Jimin Yoon, Eun Kyoung Chung, Hyeong Geun Jo, Tae-Hun Kim.

**Supervision:** Jieun Choi, Dongwoo Nam.

**Validation:** Eun-Jung Kim.

**Visualization:** Won-Suk Sung.

**Writing – original draft:** Won-Suk Sung, Yejin Hong.

**Writing – review & editing:** Eun-Jung Kim, Jieun Choi, Dongwoo Nam.
